# Discordance of pathological thin melanoma thickness and T stage in SEER registry: impacts on clinical management and research directions

**DOI:** 10.18632/oncotarget.21980

**Published:** 2017-10-24

**Authors:** Zhichao Wang, Haizhou Li, Xinyang Liu, Jinhong Bae, Xin Huang, Yashan Gao, Xiangwen Xu, Jihan Guo, Lin Lu, Tao Zan, Qingfeng Li

**Affiliations:** ^1^ Department of Plastic and Reconstructive Surgery, Shanghai Ninth People's Hospital, Shanghai Jiao Tong University School of Medicine, Shanghai, China; ^2^ Zhongshan Hospital, Fudan University, Shanghai, China

**Keywords:** melanoma, SEER, tumor thickness, T stage, discordance

## Abstract

**Background:**

Ultrathin melanoma was previously demonstrated to have higher risk for melanoma-specific mortality using SEER database. However, these guideline-changing conclusions has been recently challenged by miscoding of thickness. This present study was performed to assess the prognosis of thin and ultrathin melanoma using only surgically-treated, pathologically confirmed and after removal of discordant cases.

**Methods:**

Melanoma patients from SEER database who were initially diagnosed with histologically confirmed and surgically treated melanoma from 1998 to 2012 were included. Subjects with discordance between T stage and tumor thickness were excluded. Kaplan-Meier curves, log-rank test and multivariate Cox proportional hazards regression models were used.

**Results:**

55,754 patients met the strict inclusion criteria, but 16 (0.02%) and 803 (1.4%) patients were removed due to T0 stage and discordance between T stage and thickness, respectively. Therefore, 54,935 patients entered the analyses, among which 52,751 were LN negative and 2,184 were LN positive. In either overall or LN-negative patients, a straightforward dose-effect relationship of larger thickness with increasing mortality was observed. In contrast, in LN positive patients, the T1 subgroup demonstrated a similar survival with tumors in T2 mm subgroup. Multivariable analysis revealed same pattern, and significant interaction between T stage and LN involvement was found. Further categorizing T1 melanoma into 10 equal 0.10 mm increments demonstrated an unexpected “N”-shaped pattern of mortality in overall and LN negative ultrathin melanoma but not in LN positive melanoma.

**Conclusions:**

No difference in mortality was observed in T1-3 tumors with LN involvement. External and independent validation studies are warranted.

## INTRODUCTION

Incidence of malignant melanoma is increasing rapidly, even in countries with historically low incidence rate [1, 2]. According to the American Joint Committee on Cancer (AJCC), stage I cutaneous melanomas make up approximately 78% of all newly diagnosed cases in the United States, of which more than 80% are thin melanoma (tumor thickness less than 1.00 mm) [3]. Knowledge about disease patterns and outcomes of thin melanomas will lead to better clinical managements and benefit melanoma patients.

Large population-based cancer registries are useful tools for determining cancer outcomes. By systematically collecting, storing and reporting data on patients with specific cancer of interest, cancer registry data can provide insightful findings to guide clinical practice. Surveillance, Epidemiology and End Results (SEER) is one of the largest cancer registries worldwide and has been used frequently in thin melanoma researches [4].

Based on melanoma SEER data from 1998 to 2008, Sanjay *et al* [5] discovered that tumor thickness ≤0.50 mm was a marker for poor prognosis in the setting of positive lymph node (LN) metastasis. Even in thin melanoma without positive LN status, Shoshana *et al* [6] identified that the 10-year risk of death was higher for thin melanoma with tumor thickness 0.01–0.25 mm than those with tumor thickness from 0.26–0.50 mm. Prognosis pattern worsened with tumor thickness only starting from 0.51 mm and this unexpected pattern could not be explained by ulceration.

However, all these potentially guideline-changing findings have been challenged by significant proportion of miscoding of melanoma thickness in SEER registry, especially in thin melanoma (tumor thickness ≤ 1.00 mm). After re-examination of tumor thickness in one SEER region using pathology reports, decimal point placement was found to be the most common error in thin melanoma. After correction, 96% of original ultrathin-related death and 100% of ultrathin related positive LN status was determined to be miscoded [7].

With such significant miscoding error in thin melanoma in the SEER database, the previous findings of thin melanoma could be artificial and misleading. In order to get the most reliable subset of melanoma SEER data, firstly, we established strict inclusion/exclusion criteria to only get surgically treated, pathological confirmed melanoma entries. Secondly, parameters coded as unknown (including missing data) are treated as a separate category in the present analyses instead of “negative findings”, which was taken for granted and routinely performed by previous melanoma SEER studies. Thirdly, internal consistency checks were performed for every included melanoma entry and inconsistent entries were removed.

Our hypothesis was that loose inclusion/exclusion criteria, improper categorization of unknown parameters and data internal inconsistency might be greatly biased previous findings and mislead clinical practice. In the present study, based on the most reliable data possible, we tested whether thin and ultrathin melanoma still indicated poor prognosis, especially in different lymph node subgroups.

## RESULTS

### Baseline characteristics

55,754 patients met the strict inclusion criteria. 16 patients were excluded for T0 stage (unknown primary site) and 803 patients were further excluded for discrepancy between database derived T stage and manual categorization of tumor thickness according to AJCC criteria. In total, 54,935 patients entered the analyses, among which 52,751 were LN negative and 2,184 were LN positive.

Baseline characteristics were shown in Table 1. The included patients were dominantly white and around half were male. The median follow-up time was 48 months. Categories of LN involvement significantly differed with respect to T stage, sex, age, race, marital status, ulceration, mitogenic status and number of LNs dissected. Patients with LN-positive disease tended to have thicker primary tumors and more LNs examined than those with LN-negative disease. Expectedly, no Tis patients had LN involvement. In patients with positive LN status, the rate of primary melanoma with ulceration and positive mitogenic status was higher. It is clinically reasonable to demonstrated significantly higher number of LN dissected in LN positive patients. There was no significant difference in year of diagnosis in patients with LN-negative or LN-positive disease. The rate of LN positive patients in unmarried patients’ group (7%, 733/10,523) is 1.5 times higher than in married patients’ group (5%, 1,365/27,376).

**Table 1:** Baseline characteristics of the included subjects

Characteristics	LN negative (*N* = 52,751)	LN positive (*N* = 2,184)	Total (*N* = 54,935)	*P* value
**Median follow-up (mo, IQR)**	48 (26–75)	33 (17–62)	48 (25–75)	<0.001^#^
Year of diagnosis				0.502
1998–2004	4,878 (9.25)	218 (9.98)	5,096 (9.28)	
2005–2008	23,618 (44.77)	974 (44.60)	24,592 (44.77)	
2009–2012	24,255 (45.98)	992 (45.52)	25,247 (45.96)	
**Gender**				<0.001^*^
Male	28,585 (54.19)	1,405 (64.33)	29,990 (54.59)	
Female	24,166 (45.81)	779 (35.67)	24,945 (45.41)	
**Patient age**				<0.001^*^
18–29	2,264 (4.29)	137 (6.27)	2,401 (4.37)	
30–39	4,533 (8.59)	235 (10.76)	4,768 (8.68)	
40–49	8,415 (15.95)	386 (17.67)	8,801 (16.02)	
50–59	12,319 (23.35)	520 (23.81)	12,839 (23.37)	
60–69	12,414 (23.53)	460 (21.06)	12,874 (23.43)	
70–85	12,806 (24.28)	446 (20.42)	13,252 (24.12)	
**Race**				<0.001^*^
White	50,297 (95.35)	2,087 (95.56)	52,384 (95.36)	
Black	94 (0.18)	22 (1.01)	116 (0.21)	
Others	405 (0.77)	69 (3.16)	474 (0.86)	
Unknown	1,955 (3.71)	6 (0.27)	1,961 (3.57)	
**Marital status**				<0.001^*^
Married	26,011 (49.31)	1,365 (62.50)	27,376 (49.83)	
Not married	9,790 (18.56)	733 (33.56)	10,523 (19.16)	
Unknown	16,950 (32.13)	86 (3.94)	17,036 (31.01)	
**No. LN dissected**				<0.001^*^
≤6	51,529 (97.68)	591 (27.06)	52,120 (94.88)	
>6	973 (1.84)	1,562 (71.52)	2,535 (4.61)	
Unknown	249 (0.47)	31 (1.42)	285 (0.51)	
**Ulceration**				<0.001^*^
Yes	2,684 (88.75)	942 (43.05)	3,626 (6.51)	
No	47,526 (5.01)	1,210 (55.30)	48,736 (87.44)	
Unknown	3,340 (6.24)	36 (1.65)	3,376 (6.06)	
**Mitogenic**				<0.001^*^
Yes	3,264 (6.10)	611 (27.93)	3,875 (6.95)	
No	4,789 (8.94)	29 (1.33)	4818 (8.64)	
Unknown	45,497 (84.96)	1,548 (70.75)	47,045 (84.40)	
**T stage**				<0.001^*^
Tis	23,554 (44.65)	0 (0)	23,554 (42.88)	
T1	22,041 (41.78)	296 (13.55)	22,337 (40.66)	
T2	4,250 (8.06)	643 (29.44)	4,893 (8.91)	
T3	1,890 (3.58)	672 (30.77)	2,562 (4.66)	
T4	1,016 (1.93)	573 (26.24)	1,589 (2.89)	

The total number of included melanoma patients was 54,935, including 52,751 LN-negative melanoma patients and 2184 LN-positive melanoma patients.

LN: lymph node; mo: months; IQR: interquartile range.

^*^significant at 0.05 level.

^#^*P* for log rank test.

### Impact of T stage on melanoma specific mortality under positive/negative LN status

**Figure 1:**
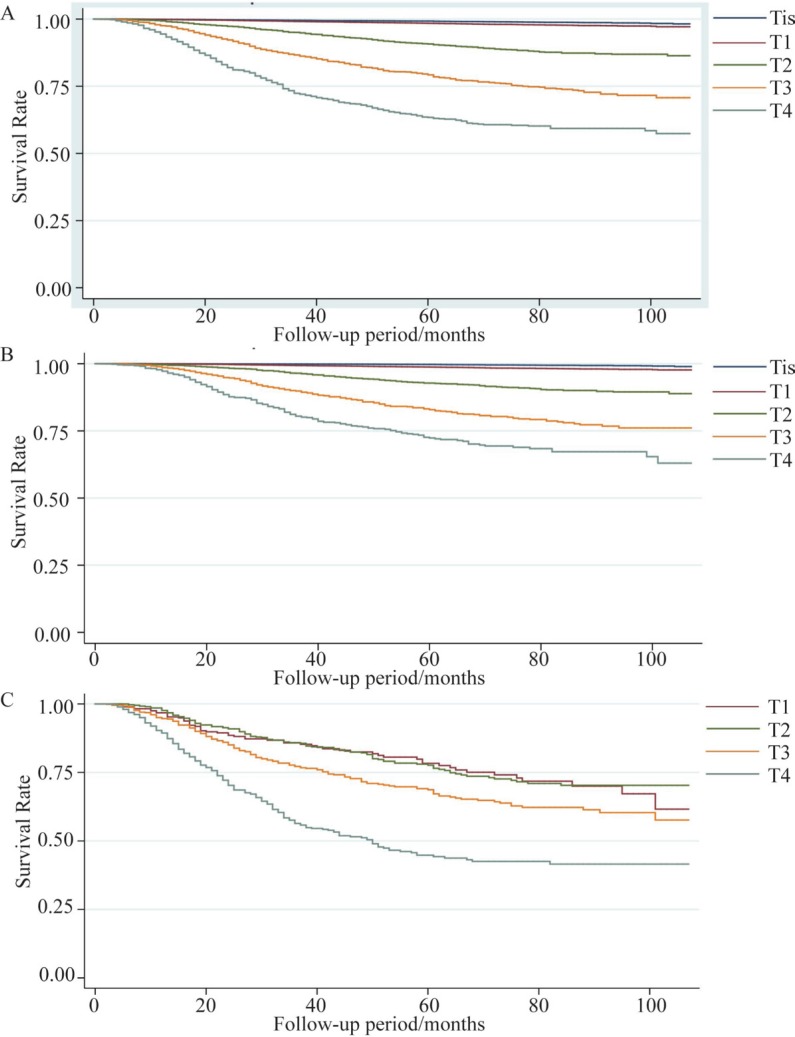
Kaplan Meier survival curves stratified by T stage (**A**) Overall number of patients: 54,935, (**B**) lymph node (LN) – negative number of patients: 52,751, and (**C**) LN – positive number of patients: 2,184 from the Surveillance, Epidemiology and End Results database were stratified into 5 categories based on AJCC T staging.

Overall and separate crude Kaplan-Meier curves for LN positive and LN negative patients stratified by T stage are provided in Figure 1. In either overall or LN-negative patients, a positive correlation of larger thickness with increasing mortality was observed. In contrast, in LN positive patients, the T1 subgroup demonstrated a similar survival (median 43 months) with tumors in T2 (median 40 months) subgroup, and the median survival in T3 and T4 subgroups were 34 and 25 months, respectively.

In multivariable analyses controlling for age, sex, year of diagnosis, marital status, ulceration, mitogenic status, lactic dehydrogenase (LDH) elevation and number of LN dissected, there was significant interaction between T stage and LN involvement (*P* < 0.001) using the likelihood ratio test, which compared the models with and without interaction terms. Each interaction term was also statistically significant in Wald test (all *P* < 0.001). In addition, the potential confounders were all independently related to melanoma specific mortality.

In the absence of LN involvement (*n* = 52,751), the hazard ratio (HR) increased monotonically with increasing tumor thickness. Compared to T1 melanoma, hazard ratios of Tis, T2, T3 and T4 groups were 0.28 (95% confidence interval (CI) 0.21–0.36, *p* < 0.001), 3.24 (95% CI 3.90–4.70, *p* < 0.001), 6.95 (95% CI 5.69–8.50, *p* < 0.001) and 10.65 (95% CI 8.60–13.20, *p* < 0.001), respectively (Table 2).

**Table 2:** Impact of T stage on melanoma specific mortality

	Overall			Pairwise		
	HR	95% CI	*P*	HR	95% CI	*P*
**Tis N0**	0.28	(0.21, 0.36)	<0.001^*^	0.28	(0.21, 0.36)	<0.001^#^
**T1 N0**	1.00	(Reference)	-	1.00	(Reference)	-
**T2 N0**	4.03	(3.35, 4.84)	<0.001^*^	3.90	(3.24, 4.70)	<0.001^#^
**T3 N0**	7.42	(6.13, 8.99)	<0.001^*^	6.95	(5.69, 8.50)	<0.001^#^
**T4 N0**	11.34	(9.25, 13.90)	<0.001^*^	10.65	(8.60, 13.20)	<0.001^#^
**Tis N+**	-	-	-	-	-	-
**T1 N+**	14.14	(10.37, 19.28)	<0.001^*^	1.00	(Reference)	-
**T2 N+**	13.31	(10.34, 17.13)	<0.001^*^	0.94	(0.69, 1.30)	0.724
**T3 N+**	15.09	(11.91, 19.13)	<0.001^*^	1.14	(0.84, 1.54)	0.403
**T4 N+**	27.78	(22.06, 34.99)	<0.001^*^	2.02	(1.50, 2.73)	<0.001^#^

The number of LN-negative melanoma patients was 52751 and the number of LN-positive patients was 2184.

HR: hazard ratio; CI: confidence interval.

^*^significant at 0.05 level.

^#^significant at 0.025 level after Borferroni correction to adjust for multiple comparison.

Among patients with LN involvement (*n* = 2,184), using T1 tumors as the reference group, hazard ratio of melanoma specific mortality remained similar in T2 tumors (HR 0.94, 95% CI 0.69–1.30, *p* = 0.724) and T3 tumors (HR 1.14, 95% CI 0.84–1.54, *p* = 0.403), and subsequently increased in T4 tumors (HR 2.02, 95% CI 1.50–2.73, *p* < 0.001).

**Figure 2:**
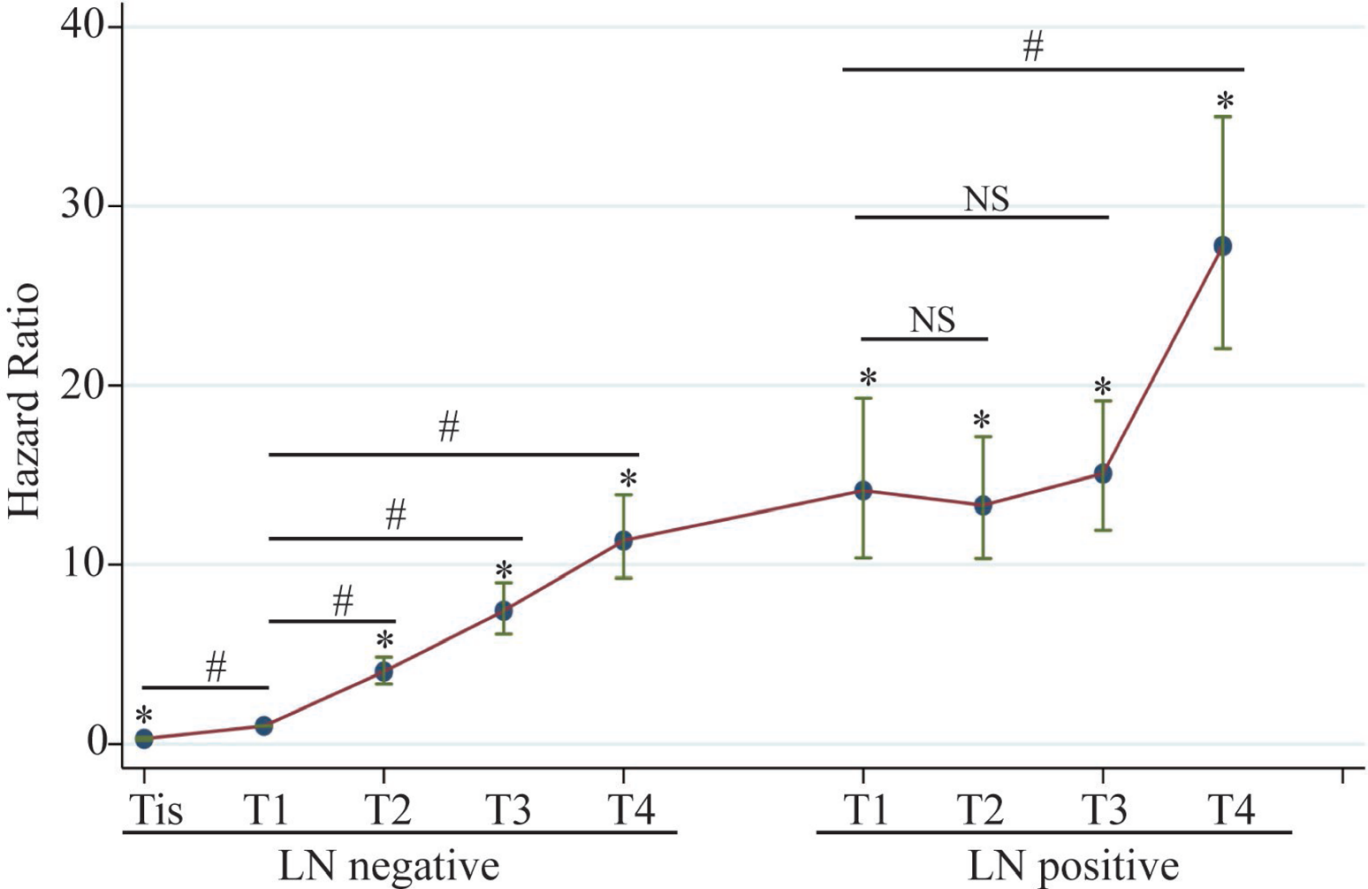
Adjusted hazard ratios for melanoma specific mortality in different combinations of LN involvement and T stage Hazard ratios were adjusted for age, sex, race, year of diagnosis, marital status, number of LN dissected, ulceration, mitogenic status and LDH elevation. Left half: LN-negative melanoma, number of patients: 52,751; Right half: LN-positive melanoma, number of patients: 2,184.

Figure 2 showed graphically the multi-adjusted hazard ratio of melanoma specific mortality in different combinations of LN involvement and T stage using T1N0M0 as reference. LN-positive tumors demonstrated worse prognosis than LN-negative tumors generally. The slope of increase in HR is sharper in LN negative than in LN positive subgroup.

### Impact of tumor thickness on melanoma specific mortality under positive/negative LN status in ultrathin melanoma

**Figure 3:**
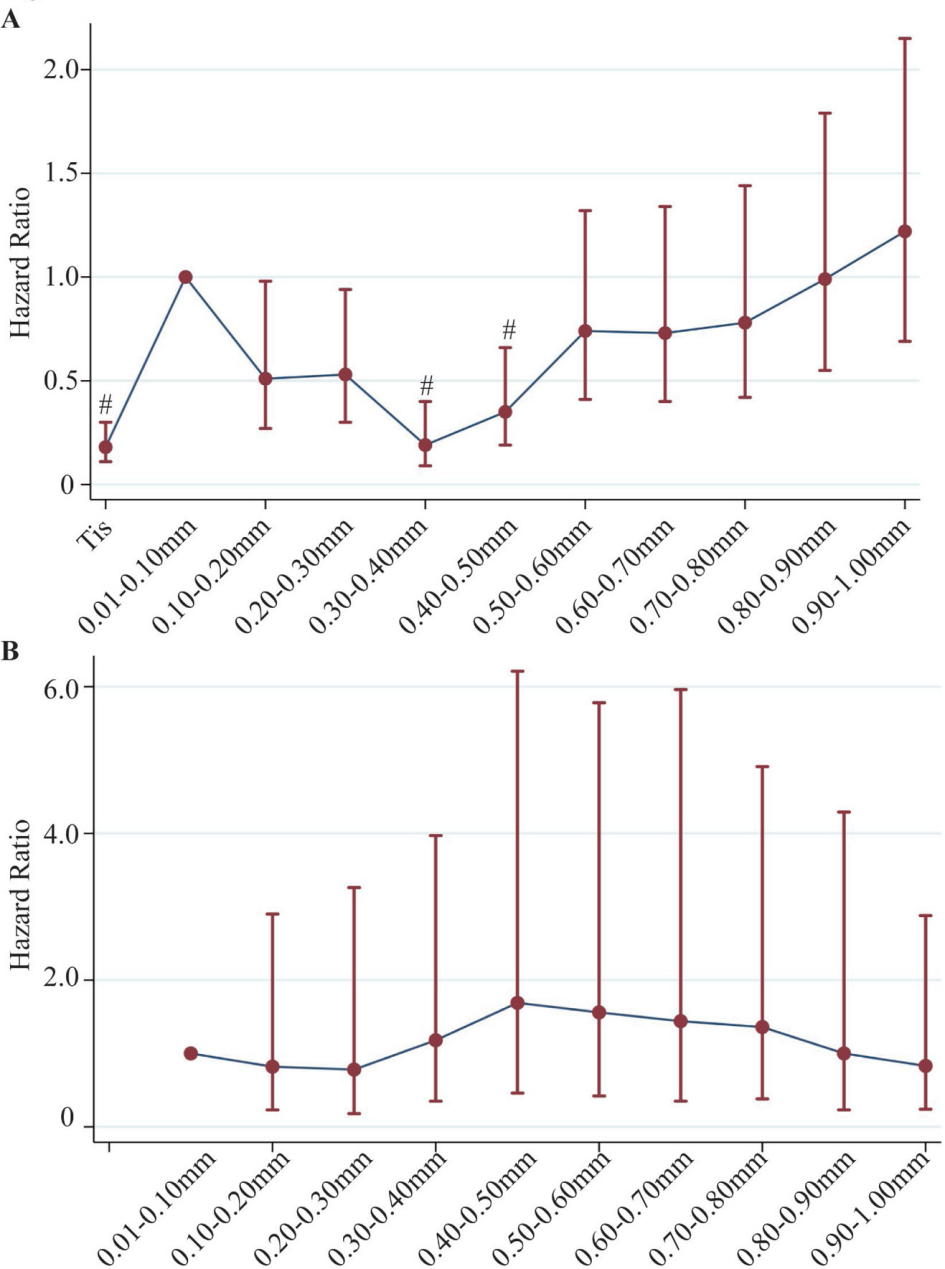
Adjusted hazard ratios for melanoma specific mortality in different combinations of LN involvement and thickness in ultrathin melanoma (**A**) Under negative LN involvement, including Tis and 10 equal 0.10 mm increments, using 0.01–0.10 mm group as reference group; Number of patients: Tis+T1, 45,595 patients; (**B**) Under positive LN involvement, including 10 equal 0.10 mm increments, using 0.01–0.10 mm group as reference group; Number of patients: T1, 296 patients. Hazard ratios were adjusted for age, sex, race, year of diagnosis, marital status, number of LN dissected, ulceration, mitogenic status and LDH elevation.

In order to account for potential residual confounding in the categorization of T1 stage, we further categorized T1 melanoma into 10 equal 0.10 mm increments (0.01–0.10 mm, 0.11–0.20 mm, …, and 0.91–1.00 mm). Melanoma specific morality was then compared in ultrathin melanoma, namely Tis and T1 subgroups (Table 3, Figure 3).

**Table 3:** Impact of tumor thickness on melanoma specific mortality in ultrathin melanoma

		Overall				LN negative				LN positive			
		No.	HR	95% CI	*P*	No.	HR	95% CI	*P*	No.	HR	95% CI	*P*
Tis	Tis	23,544	0.21	(0.13, 0.34)	<0.001^*^	23,544	0.18	(0.11, 0.30)	<0.001^#^	0			
T1	0.01–0.10 mm	799	1.00	(Reference)	-	762	1.00	(Reference)	-	17	1.00	(Reference)	-
	0.10–0.20 mm	1,877	0.67	(0.38, 1.18)	0.163	1,848	0.51	(0.27, 0.98)	0.042	29	0.82	(0.23, 2.90)	0.755
	0.20–0.30 mm	3,898	0.64	(0.38, 1.08)	0.096	3,877	0.53	(0.30, 0.94)	0.029	21	0.78	(0.18, 3.26)	0.737
	0.30–0.40 mm	3,800	0.30	(0.16, 0.55)	<0.001^*^	3,781	0.19	(0.09, 0.40)	<0.001^#^	19	1.18	(0.35, 3.97)	0.790
	0.40–0.50 mm	3,455	0.53	(0.31, 0.93)	0.028^*^	3,428	0.35	(0.19, 0.66)	0.001^#^	27	1.69	(0.46, 6.21)	0.433
	0.50–0.60 mm	2,533	0.93	(0.55, 1.59)	0.797	2,510	0.74	(0.41, 1.32)	0.305	23	1.56	(0.42, 5.78)	0.503
	0.60–0.70 mm	1,947	0.94	(0.54, 1.63)	0.815	1,920	0.73	(0.40, 1.34)	0.314	27	1.44	(0.35, 5.96)	0.614
	0.70–0.80 mm	1,658	0.97	(0.56, 1.68)	0.918	1,624	0.78	(0.42, 1.44)	0.435	34	1.36	(0.38, 4.91)	0.635
	0.80–0.90 mm	1,296	1.06	(0.62, 1.83)	0.823	1,259	0.99	(0.55, 1.79)	0.970	37	1.00	(0.23, 4.29)	0.996
	0.90-1.00 mm	1,094	1.24	(0.75, 2.06)	0.408	1,032	1.22	(0.69, 2.15)	0.503	62	0.83	(0.24, 2.88)	0.765

The total number of Tis and T1 melanoma patients was 45891; The number of LN-negative Tis and T1 patients was 45595; The number of LN-positive T1 patients was 296.

HR: hazard ratio; CI: confidence interval.

^*^significant at 0.05 level, reference group T1 0.01–0.10 mm group.

^#^significant at 0.025 level after Borferroni correction of 10 to adjust for multiple comparison, reference group T1 0.01–0.10 mm group.

Within T1, the distribution of tumor thickness was generally even. Among these ultrathin Tis and T1 subcategories, we did not find a similar pattern of worse prognosis with greater thickness. The thinnest Tis subgroup had the best survival compared to 0.01–0.10 mm subgroup (HR 0.21, 95%CI 0.13–0.34, *p* < 0.001). However, an unexpected trend of decrease in HR was observed in the next two subgroups with a thickness of 0.11–0.30 mm, and reached another bottom at 0.31–0.40 mm and 0.41–0.50 mm subgroups, which were significantly lower than the reference group. Subsequently, a gradual increase in HR was observed in the last 5 subgroups without statistically significant difference. A similar pattern was observed in LN negative melanomas. In LN positive melanomas, no obvious difference in hazard of melanoma specific mortality was observed with a limited number of subjects in each subgroup. The pattern could not be clearly explained by ulceration status (Supplementary Table 1). Re-categorization into 4 equal 0.25 mm increments revealed similar patterns (Supplementary Table 2). Dichotomizing T1 into 0.01–0.50 mm and 0.51–1.00 mm also did not show difference in mortality in LN positive patients. (Supplementary Table 3).

## DISCUSSION

Although the traditional view of malignant progression is that cancer gains metastatic ability through an accumulation of mutations as they grow to a large size, recent studies have suggested that for some tumors, the acquisition of metastatic potential may occur early in cancer development, even in the absence of detectable primary tumors. It was previously reported that very small tumor size with intensive lymph node involvement was related to worse survival in breast cancer [8], colon cancer [9] and prostate cancer [10]. In melanoma, Sanjay *et al* [5] discovered that in LN positive melanomas, patients with tumor thickness ≤0.50 mm had higher mortality than those with tumor thickness 0.51–1.00 mm or 1.01–2.00 mm. More unexpectedly, Shoshana *et al* [6] recently identified that in LN negative melanoma, patients with tumor thickness 0.01–0.25 mm had higher mortality than those with tumor thickness 0.26–0.50 mm. In our study, we did demonstrate the effect modification by LN involvement in the effect of T stage on melanoma specific mortality. In LN positive melanoma, T stage became a less important predictor of mortality than in the LN negative setting. However, in LN positive setting, we did not find a worse prognosis in patients with T1 melanoma than those with higher T stage.

Residual confounding remains an important issue when categorizing continuous variables into categorical variables. Both Sanjay *et al* [5] and Shoshana *et al* [6] further categorized T1 stage into smaller subgroups according to tumor thickness. Repeating their analyses yielded different results. More specifically, when dichotomizing T1 into 0.01–0.50 mm and 0.51–1.00 mm like what Sanjay *et al* [5] did, our data did not show a higher mortality in 0.01–0.50 mm subgroup compared to 0.51–1.00 mm and T2 subgroups. A potential explanation is the difference in study population and misclassification bias. Sanjay *et al* included 6,134 LN positive subjects in 1998–2008 database, while we only could include 2,184 LN positive subjects in 1998–2012 database based on our stricter inclusion criteria which only got surgically treated and pathological confirmed melanoma entries, minimizing misclassification. More intensive categorization into 4 or 10 categories did not identify difference in mortality in LN positive melanoma either.

Re-categorizing T1 into 4 categories mimicking Shoshana *et al*'s method [6] showed similar results with Shoshana *et al*. However, Shoshana did not take Tis into account when looking the relationship of subgroups in T1 and mortality in LN negative melanoma. When adding the subgroup of Tis, the sharp increase in mortality from Tis to 0.01–0.25 mm made the results even harder to interpret. In view of the large sample size, we further categorized T1 into 10 categories. The analyses together with Tis subgroup confirmed the same pattern that the mortality increased sharply from Tis to 0.01–0.10 mm, then decreased to bottom at 0.31–0.50 mm, and then increased afterwards. Although some P values were statistically significant due to large sample size, the “N”-shaped pattern of mortality within a very small range of thickness was not biologically plausible.

Recently, Gimotty *et al* [7] reported significant proportion of miscoding of melanoma thickness in SEER registry, especially in thin melanoma (tumor thickness ≤1.00 mm). After re-examination of tumor thickness in one SEER region using pathology reports, decimal point placement was found to be the most common error in thin melanoma. After correction, 96% of original ultrathin-related death and 100% of ultrathin related positive LN status were determined as miscoded [7]. Similar coding errors in very small tumor size were proved in prostate cancer [11]. Furthermore, the miscoding of tumor thickness was also observed in our analyses. Notably, 803 subjects were excluded because of discrepancy between database derived T stage and manual categorization of tumor thickness according to AJCC criteria. Among them, 119 patients were recorded to have a thickness of 0 mm while T1-T4. Contrarily, 485 patients recorded as Tis had a thickness of more than 0.01 mm, most of which (436, 90%) of which were in the 0.01–0.10 mm category. Although our internal consistency checks had removed some of the coding errors, we have reasons to believe that still considerable coding errors exist even in those concordant entries. As in the code for tumor thickness, “1” but not “0.01” representing 0.01 mm, miscoding was easier to occur in ultrathin melanoma. Based on our analyses and previous literatures, we believe coding error or errors in the source document should explain part of the unexpected pattern of mortality in ultrathin melanoma. Therefore, a more standardized process should be established for SEER data collection and training specialized personal for SEER data entry is highly recommended at each SEER data contributing set [12, 13]. In addition, it might be useful to retrieve original medical documents and double check data entries of melanoma patients with potential coding error.

Several limitations should be considered when interpreting our results. First, the SEER database lacks important information on cancer therapy and patient outcome variables. Thus, our analyses could not adjust for these potential confounders, for example, the undertreatments of patients with small tumors even in node-positive status and new treatment approaches developed over time. To minimize the confounding by different treatment methods in our relatively long study period, we adjusted for a surrogate confounder, year of diagnosis, and our results remained similar. Second, despite a large initial study population, individual subgroups became small after stratifying by tumor thickness and LN involvement, yielding limited statistical power. Third, coding error and misclassification were possible within the SEER database.

In conclusion, there was effect modification by LN involvement in the effect of T stage on melanoma specific mortality. No difference in mortality was observed in T1-3 tumors with LN involvement. The pattern of mortality in ultrathin melanoma was unclear and biased by coding errors. Further SEER studies should be conducted in subsequent data to see if patterns are changed and external and independent validation studies are warranted. Systematic review and meta-analysis is also valuable when sufficient data is available.

## MATERIALS AND METHODS

### Cutaneous melanoma patient selection

We obtained and analyzed data from the National Cancer Institute's SEER database after signing the SEER data-use agreement in December 2016.

Patients diagnosed after 2012 were excluded to ensure a minimum follow-up of 4 years. Patients diagnosed before 1998 were excluded because of lack of information on tumor thickness. Inclusion criteria were as follows: aged 18–85 years at diagnosis; treated with surgery; diagnosed with histologically confirmed melanoma; diagnosis not obtained through death certificate or autopsy; without distant metastasis at diagnosis; melanoma as first and only cancer diagnosis; known diagnosis date; known N stage; known T stage; and known tumor thickness. Exclusion criteria were: T0; and discrepancy between database derived T stage and manual categorization of tumor thickness according to AJCC criteria (Tis 0.00 mm; T1, 0.01–1.00 mm; T2, 1.01–2.00 mm; T3, 2.01–4.00 mm; T4, >4.00 mm) [14].

Internal consistency was performed by calculating tumor thickness into T stage based on TNM staging guideline. Calculated T stage was checked with corresponding T stage in the SEER database, the patient data will be excluded if discordant finding was noted (Supplementary Figure 1).

### Outcome measures

The primary study outcome was melanoma specific mortality. Vital status code established whether the patient was alive or dead. Cause of death was categorized as melanoma specific or non-melanoma–related death. Melanoma specific mortality was calculated from the date of diagnosis to the date of melanoma death. Patients who died from other causes were censored at date of death.

### Statistical analyses

Degree of LN involvement dichotomized as LN negative or LN positive. Baseline characteristics were compared using *t*-test and Chi-square test. Kaplan-Meier methods, adjusted Cox proportional hazards models with were performed. Potential confounders included age, sex, race, year of diagnosis, marital status, number of LN dissected, ulceration, mitogenic status and LDH elevation. All missing/unknown data are listed and analyzed as unknown status. Overall presence of interaction between tumor thickness and LN involvement was evaluated by the likelihood ratio test comparing models with and without interaction terms. Wald tests were performed to evaluate specific interaction terms. Overall comparison of melanoma specific mortality was compared among different combinations of tumor thickness and LN involvement. Pair-wise comparisons were performed to compare different tumor thickness groups in both LN positive and negative patients. All computed *P* values were two sided. For the pairwise comparison in LN-positive and LN-negative subgroups, a Bonferroni correction of n-1 was applied in order to adjust for multiple comparisons, and statistical significance was defined as a *P* value lower than .025. All statistical analyses were performed using Stata 14.0. This study was submitted and determined to qualify for institutional review board exemption.

## SUPPLEMENTARY MATERIALS FIGURE AND TABLES



## Abbreviations

AJCC: American Joint Committee on Cancer
SEER: Surveillance
LN: lymph node
LDH: lactic dehydrogenase
HR: hazard ratio
CI: confidence interval.
