# Supplementary material for: Discordance of pathological thin melanoma thickness and T stage in SEER registry: impacts on clinical management and research directions
Source: Oncotarget. 2017 Oct 24;8(58):97727–35. doi: 10.18632/oncotarget.21980 (PMC5716686; doi:10.18632/oncotarget.21980)
Supplement: Supplementary file 1 [file oncotarget-08-97727-s001.pdf]

Discordance of pathological thin melanoma thickness and T stage in SEER registry: impacts on clinical management and research directions

SUPPLEMENTARY MATERIALS

Supplementary Table 1: Impact of tumor thickness on melanoma specific mortality in ultrathin LN negative melanoma according to ulceration status

		With ulceration				Without ulceration			
		No.	HR	95% CI	*P*	No.	HR	95% CI	*P*
Tis	Tis	126	0.33	(0.45, 0.78)	0.011#	21,255	0	0	>0.999
T1	0.01-0.10 mm	52	1.00	(Reference)	-	663	1.00	(Reference)	-
	0.10-0.20 mm	57	0.65	(0.24, 1.80)	0.409	1,722	0.49	(0.19, 1.26)	0.139
	0.20-0.30 mm	69	0.85	(0.35, 2.09)	0.728	3,715	0.67	(0.27, 1.64)	0.380
	0.30-0.40 mm	60	0.18	(0.05, 0.59)	0.005#	3,628	0.35	(0.13, 0.94)	0.037
	0.40-0.50 mm	69	0.61	(0.24, 1.58)	0.309#	3,273	0.19	(0.05, 0.68)	0.011#
	0.50-0.60 mm	64	1.33	(0.54, 3.27)	0.530	2,377	0.55	(0.20, 1.57)	0.265
	0.60-0.70 mm	66	1.45	(0.58, 3.64)	0.418	1,811	0.19	(0.04, 0.85)	0.030
	0.70-0.80 mm	71	1.90	(0.77, 4.71)	0.167	1,504	0.13	(0.03, 0.58)	0.007#
	0.80-0.90 mm	79	2.64	(1.07, 6.54)	0.035	1,143	0.21	(0.07, 0.66)	0.007#
	0.90-1.00 mm	80	3.30	(1.35, 8.13)	0.009#	916	0.38	(0.15, 0.92)	0.033

The number of included Tis and T1 patients with ulceration was 793; the number of included Tis and T1 patients without ulceration was 42007.

HR: hazard ratio; CI: confidence interval.

#significant at 0.025 level after Borferroni correction of 10 to adjust for multiple comparison, reference group T1 0.01–0.10 mm group.

Supplementary Table 2: Impact of tumor thickness (0.25 mm increments) on melanoma specific mortality in ultrathin melanoma

		LN negative				LN positive			
		**No.**	**HR**	**95% CI**	***P***	**No.**	**HR**	**95% CI**	***P***
**Tis**	**Tis**	23,554	0.25	(0.18, 0.36)	<0.001[#]	0			
**T1**	**0.01-0.10 mm**	4,277	1.00	(Reference)	-	56	1.00	(Reference)	-
	**0.10-0.20 mm**	9,419	0.40	(0.28, 0.60)	<0.001[#]	57	1.34	(0.62, 2.89)	0.453
	**0.20-0.30 mm**	5,139	1.10	(0.78, 1.56)	0.601	63	1.71	(0.74, 3.92)	0.209
	**0.30-0.40 mm**	3,206	1.38	(0.97, 1.98)	0.074	120	1.01	(0.45, 2.27)	0.975

The number of LN-negative Tis and T1 melanoma patients was 45595; the number of LN-positive T1 patients was 296.
HR: hazard ratio; CI: confidence interval; LN, lymph node;
[#]significant at 0.025 level after Borferroni correction of 4 to adjust for multiple comparison, reference group T1 0.01–0.10 mm group.

Supplementary Table 3: Impact of tumor thickness (0.50 mm increments) on melanoma specific mortality

	LN negative				LN positive			
	**No.**	**HR**	**95% CI**	***P***	**No.**	**HR**	**95% CI**	***P***
**Tis**	23,554	0.40	(0.29, 0.54)	<0.001[#]	0			
**T1 0.01–0.50 mm**	13,696	1.00	(Reference)	-	113	1.00	(Reference)	-
**T1 0.51–1.00 mm**	8,354	2.12	(1.64, 2.74)	<0.001[#]	183	0.83	(0.49, 1.39)	0.475
**T2**	4,250	5.62	(4.43, 7.14)	<0.001[#]	643	0.86	(0.57, 1.29)	0.455
**T3**	1,089	10.05	(7.83, 12.89)	<0.001[#]	672	1.03	(0.69, 1.54)	0.873
**T4**	1,016	15.41	(11.87, 20.00)	<0.001[#]	573	1.83	(1.24, 2.71)	0.002[#]

The number of LN-negative Tis and T1 melanoma patients was 45595; the number of LN-positive T1 patients was 296.
HR: hazard ratio; CI: confidence interval; LN, lymph node.
[#]significant at 0.025 level after Borferroni correction of 4 to adjust for multiple comparison, reference group T1 0.01–0.10 mm group.

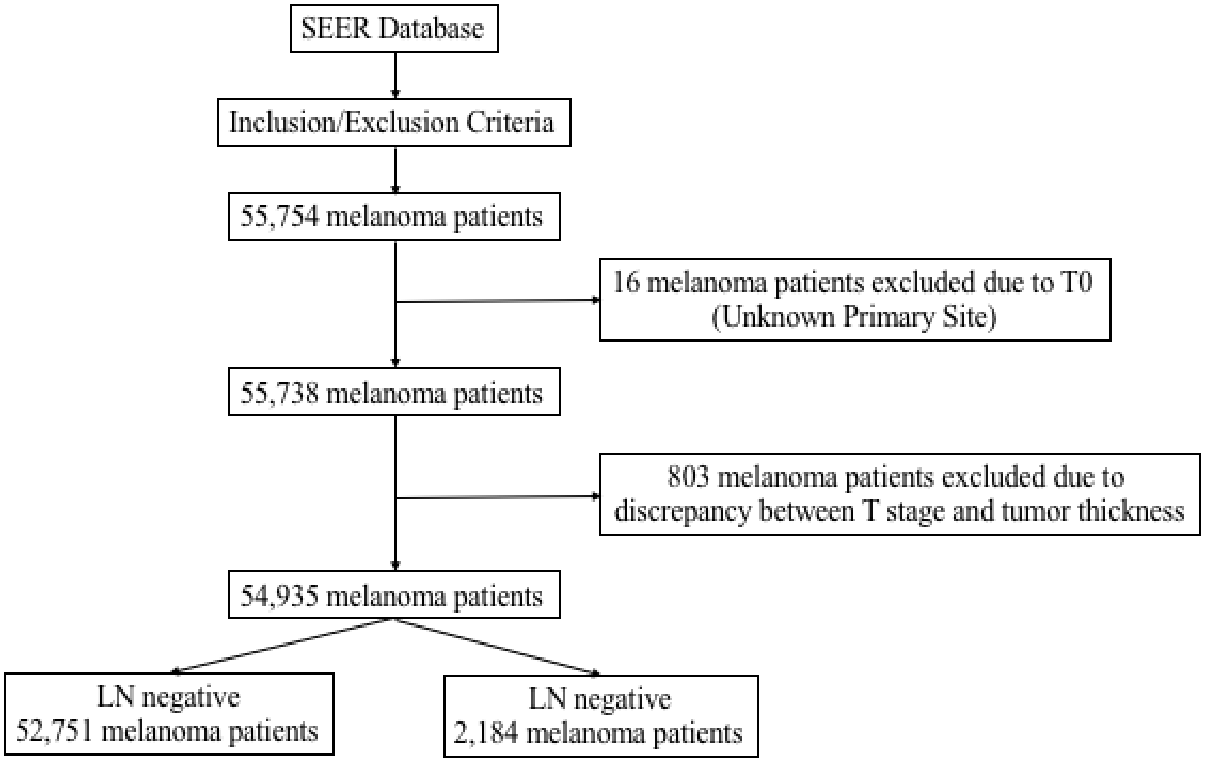

Supplementary Figure 1: Flow diagram of patient selection in SEER database.
